# Predicting the effect of different folate doses on [^68^Ga]Ga-PSMA-11 organ and tumor uptake using physiologically based pharmacokinetic modeling

**DOI:** 10.1186/s13550-023-01008-y

**Published:** 2023-06-15

**Authors:** Hinke Siebinga, Jeroen J. M. A. Hendrikx, Alwin D. R. Huitema, Berlinda J. de Wit-van der Veen

**Affiliations:** 1 Department of Pharmacy and Pharmacology, The Netherlands Cancer Institute, Amsterdam, The Netherlands; 2 Department of Nuclear Medicine, The Netherlands Cancer Institute, Amsterdam, The Netherlands; 3 Department of Clinical Pharmacy, University Medical Center Utrecht, Utrecht University, Utrecht, The Netherlands; 4 Department of Pharmacology, Princess Máxima Center for Pediatric Oncology, Utrecht, The Netherlands

**Keywords:** [^68^Ga]Ga-PSMA-11, Folate, Folic acid, PSMA, PBPK model

## Abstract

**Background:**

Folate intake might reduce [^68^Ga]Ga-PSMA-11 uptake in tissues due to a competitive binding to the PSMA receptor. For diagnostic imaging, this could impact decision making, while during radioligand therapy this could affect treatment efficacy. The relationship between folate dose, timing of dosing and tumor and organ uptake is not well established. The aim of this study was to develop a physiologically based pharmacokinetic (PBPK) model to predict the effect of folates on [^68^Ga]Ga-PSMA-11 PET/CT uptake in salivary glands, kidneys and tumors.

**Methods:**

A PBPK model was developed for [^68^Ga]Ga-PSMA-11 and folates (folic acid and its metabolite 5-MTHF), with compartments added that represent salivary glands and tumor. Reactions describing receptor binding, internalization and intracellular degradation were included. Model evaluation for [^68^Ga]Ga-PSMA-11 was performed by using patient scan data from two different studies (static and dynamic), while for folates data from the literature were used for evaluation. Simulations were performed to assess the effect of different folate doses (150 µg, 400 µg, 5 mg and 10 mg) on accumulation in salivary glands, kidney and tumor, also for patients with different tumor volumes (10, 100, 500 and 1000 mL).

**Results:**

Final model evaluation showed that predictions adequately described data for both [^68^Ga]Ga-PSMA-11 and folates. Predictions of a 5-MTFH dose of 150 µg and folic acid dose of 400 µg (in case of administration at the same time as [^68^Ga]Ga-PSMA-11 (*t* = 0)) showed no clinically relevant effect on salivary glands and kidney uptake. However, the effect of a decrease in salivary glands and kidney uptake was determined to be clinically relevant for doses of 5 mg (34% decrease for salivary glands and 32% decrease for kidney) and 10 mg (36% decrease for salivary glands and 34% decrease for kidney). Predictions showed that tumor uptake was not relevantly affected by the co-administration of folate for all different folate doses (range 150 µg–10 mg). Lastly, different tumor volumes did not impact the folate effect on [^68^Ga]Ga-PSMA-11 biodistribution.

**Conclusion:**

Using a PBPK model approach, high doses of folate (5 and 10 mg) were predicted to show a decrease of [^68^Ga]Ga-PSMA-11 salivary glands and kidney uptake, while intake by means of folate containing food or vitamin supplements showed no relevant effects. In addition, tumor uptake was not affected by folate administration in the simulated dose ranges (150 µg–10 mg). Differences in tumor volume are not expected to impact folate effects on [^68^Ga]Ga-PSMA-11 organ uptake.

**Supplementary Information:**

The online version contains supplementary material available at 10.1186/s13550-023-01008-y.

## Introduction

The prostate-specific membrane antigen (PSMA) receptor is abundantly expressed on the cell surface of nearly all prostate cancer (PCa) cells [1]. Targeting the PSMA receptor using radiolabeled PSMA ligands proved a valuable strategy for both diagnostic imaging of PCa as well as treatment in the advanced setting. Despite its name, PSMA (or glutamate carboxypeptidase II [GCP II]) was also identified in various other tissues, such as the small intestine, kidney nephrons and salivary glands [2, 3]. The active target of a PSMA receptor consists of two binding sites, namely the glutamate-sensing pocket and a lipophilic binding pocket (arene-binding site), and the affinity of ligands for the receptor is increased after addressing both binding sites [4–6]. Folates (including polyglutamates, monosodium glutamate (MSG), folic acid and 5-methyltetrahydratefolate) contain a glutamate structure and also target the arene-binding site, and consequently, folates could act as a competitor by blocking the binding of the PSMA radioligands [7–9]. For diagnostic imaging, the potential effect of folates on the biodistribution could impact decision making, while during radioligand therapy this could even affect treatment efficacy.

The putative effect of folate intake on the biodistribution of diagnostic PSMA ligands has been demonstrated in two small prospective studies [10, 11]. Harsini et al*.* and Armstrong et al*.* found that MSG administration (12.7 g and 150 mg/kg for the respective studies) prior to gallium-68 (^68^Ga) PSMA-11 or fluorine-18 (^18^F) DCFPyL administration lowered tracer uptake in salivary glands and tumor lesions significantly [10, 11]. Apart from having an undesirable effect on imaging accuracy, folate co-administration has also been suggested as a potential approach to intentionally reduce organ uptake, and subsequent toxicity, during PSMA-based radionuclide therapy [12]. As a first attempt applying this approach, Sarnelli et al*.* showed that administration of folic polyglutamate indeed significantly reduced lutetium-177 (^177^Lu) PSMA-617 uptake in salivary glands compared to previous dosimetry evaluations, though effects on tumor uptake were not assessed [13]. Although extrapolation from diagnostic to therapeutic results is challenging, diagnostic tumor uptake was reduced after folate administration, which indicated limited benefits for this approach in clinical practice due to potential decreased treatment efficacy [10, 11]. However, high doses of MSG (12.7 g and 150 mg/kg) were administered to patients in these studies and lower folate doses could potentially achieve saturation on organ tissue without affecting tumor uptake. Therefore, it is crucial to evaluate the effect of different folate doses and timing of these doses on biodistribution, which is also important since folates are ingested in low quantities daily and are found in many vitamin supplements [11, 14]. In addition, considering a tumor sink effect, a greater impact of folate administration on tumor uptake in patients with a high tumor burden might be expected [11].

To study the effects of folate intake and timing of intake on PSMA radioligand distribution, a physiologically based pharmacokinetic (PBPK) modeling approach was used. Main advantages of this noninvasive approach are that predictions of folate effects can easily be extrapolated to different clinical scenarios (e.g., comparing patients with different tumor volumes) and subsequent prospective trials are either not required or can efficiently be informed by the results [15]. Hence, the aim of this study was to predict the effect of different doses of folate administrations (representing both folate containing food intake, vitamin supplements and high-dose folic acid administration) prior to [^68^Ga]Ga-PSMA-11 PET/CT on uptake in salivary glands, kidney and tumors using PBPK modeling. In addition, the effect of different timings of folate intake and increasing tumor volume on the impact of folate administration on [^68^Ga]Ga-PSMA-11 biodistribution was determined. Gained information based on these simulations could then guide future trial design or decision making for the use of folates in clinical practice.

## Methods

### PBPK model development

A drug–drug interaction approach was used to include the competitive binding of folate and ^68^Ga-Glu-urea-Lys(Ahx)-HBED-CC ([^68^Ga]Ga-PSMA-11) to the PSMA receptors. The PBPK model for PCa patients was developed in PK-Sim® and MoBi® (Open Systems Pharmacology software, version 11.0) [16]. The model structure consisted of a standard multi-compartment model (including kidneys) provided by the software, while compartments for salivary glands, (primary) tumor and tumor metastases were manually added. Salivary glands input parameter information was based on literature values [17, 18]. For PCa lesions, the tumor volume was fixed to 9.5 mL for the primary compartment and 100 mL for all metastases combined, to represent a typical advanced PCa patient [19, 20]. Fraction interstitial and fraction vascular of these compartments were fixed to 0.38 and 0.05, respectively [17, 21]. Initial tumor blood flow and PSMA receptor expressions were based on a PBPK model for PSMA ligands published by Begum et al*.* [17]. PSMA expression for the metastases compartment was set to a value 1.4-fold higher compared to primary tumors [17, 22].

Both folate (by means of folic acid) and [^68^Ga]Ga-PSMA-11 were added as compounds, and thus, drug-specific information for both compounds was added separately. All information regarding the folate model development is provided in Additional file 1. For [^68^Ga]Ga-PSMA-11, the molecular weight was 1011.9 g/mol and lipophilicity was − 3.8 [23, 24]. Plasma protein binding for [^68^Ga]Ga-PSMA-11 was set to 57%, which was reported for [^177^Lu]Lu-PSMA-617 [25]. Renal clearance was manually scaled to 14% unchanged excretion in urine [26]. For [^68^Ga]Ga-PSMA-11, mechanisms of peptide-specific distribution and uptake (including receptor binding, internalization and intracellular degradation) were included similarly to a previously developed PBPK model for [^68^Ga]Ga-DOTATATE, which has a similar mechanism of distribution [27]. Affinity parameters were based on previously developed PBPK models for PSMA ligands [17, 28]. The injected dose for [^68^Ga]Ga-PSMA-11 was fixed to 2.49 µg, which was the median injected peptide amount in our previous research (n = 362) [19]. A built-in Monte Carlo algorithm was used for parameter identification to optimize selected input parameters based on observed patient data. Most relevant initial and optimized input parameter values are provided in Table 1.

**Table 1:** Compound-specific and system-specific parameters that were fixed or fitted to describe [^68^Ga]Ga-PSMA-11 biodistribution using the PBPK model

	Fixed or fitted (*) value	References
Molecular weight [^68^Ga]Ga-PSMA-11	1011.9 g/mol	[24]
Lipophilicity [^68^Ga]Ga-PSMA-11	− 3.8	[23]
Fraction unbound	0.43	[25]
*K*_D_	0.06 nmol/L	[28]
*k*_off_	0.015 min^−1^	[28]
*k*_int_ tumor	0.001 min^−1^	[17]
*k*_int_ other tissue	0.035 min^−1^	[28]
*k*_deg_ tumor	0.00014 min^−1^	[17]
*k*_deg_ other tissue	0.00037 min^−1^	[17]
PSMA amount tumor	0.437 nmol	[17]
PSMA amount salivary gland	0.162 nmol*	[17]
PSMA amount liver	4.00 nmol*	[17]
PSMA amount kidney	4.64 nmol*	[17]
Blood flow tumor	16.4 mL/min/100 g*	[17, 21]

* represents fitted parameter values. *K*_D_, equilibrium dissociation constant; *k*_off_, dissociation rate constant; *k*_int_: internalization rate; *k*_deg_, degradation rate; PSMA, prostate-specific membrane antigen

### Model evaluation

[^68^Ga]Ga-PSMA-11 model predictions were evaluated based on data retrieved from patients that received a [^68^Ga]Ga-PSMA-11 PET/CT in our hospital. Datasets from previously performed studies were combined for model evaluation and parameter optimization [19, 29]. The prospective trial was approved by the Antoni van Leeuwenhoek Medical Ethics Committee (NL8263) [29], while the retrospective study was approved by the Institutional Review Board of the Netherlands Cancer Institute (IRBd20-201) [19]. Detailed information regarding data acquisition and quantitative analysis of scans is provided in the published articles [19, 29]. Uptake in organs and tumors was decay corrected to time of injection, and subsequent concentrations (µg/L) were calculated based on the radioactivity concentrations (MBq/L) and administered specific activities (MBq/µg). Model evaluation was performed by visual assessment of prediction plots compared to observed data points. In addition, physiological plausibility of optimized parameters was assessed. A sensitivity analysis was performed to calculate the sensitivity of the model output for certain parameter assumptions, according to a previously published approach [27].

### Effect of folate intake on [^68^Ga]Ga-PSMA-11 accumulation

Using the final PBPK model, simulations were performed to determine the effect of folate intake prior to [^68^Ga]Ga-PSMA-11 PET/CT on uptake in organs and tumors. Folate intake comprised oral intake of both folic acid and its main metabolite 5-methylhydrofolate (5-MTHF), of which more detailed information is provided in Additional file 1. Folic acid intake represented administration via oral supplements, while 5-MTHF intake represented consumption of folate containing food.

The affinity of both folic acid and 5-MTHF for the PSMA receptor is not exactly known. Therefore, affinity parameters were based on N-acetylaspartylglutamate (NAAG) affinity for the PSMA receptor and assumed similar for both folic acid and 5-MTHF as the molecular structure of the binding moiety of all three compounds is comparable [8, 30–32]. The dissociation rate constant (*k*_off_) was fixed to 0.6 min^−1^ [32], and this *k*_off_ and literature values for the Michaelis constant (*K*_m_) and first-order turnover number (*k*_cat_) were used to calculate the dissociation constant (*K*_D_). Since published affinity values of NAAG for PSMA differed considerably, three different *K*_D_ values were taken into account in model predications, representing the minimum, maximum as well as the mean of all values that were reported [8, 30, 31]. This resulted in input values for *K*_D_ of 1 nM, 5.2 nM and 10.8 nM. The interaction between folates and [^68^Ga]Ga-PSMA-11 was assumed a competitive binding to the arene-binding pocket of the PSMA receptor [4, 5, 7, 8].

Three different doses of folic acid administration were evaluated, namely 400 µg, 5 mg and 10 mg. A dose of 400 µg folic acid represents vitamin supplement intake [33]. Also, the effect of 150 µg 5-MTHF administration was examined, which represents a folate-rich meal [14]. In addition, different intake moments for folates were simulated, namely at the time of [^68^Ga]Ga-PSMA-11 administration (*t* = 0) and 4 and 12 h before [^68^Ga]Ga-PSMA-11 administration. For the simulations of the folate effect, a prediction interval was included, based on the differences in *K*_D_ of folate for the PSMA receptor. Lastly, the impact of folate on [^68^Ga]Ga-PSMA-11 accumulation was assessed for different tumor volumes (10 mL, 100 mL, 500 mL and 1000 mL). In all cases, the effect of folate on [^68^Ga]Ga-PSMA-11 accumulation was determined at time of PET/CT scan (60 min post-injection) and was calculated as the relative difference (%) in concentration (µg/L), by dividing the difference in concentration with and without folate administration (µg/L) by the concentration in the case of no folate administration (µg/L).

Clinical relevance of accumulation changes was determined based on repeatability coefficients. A value below the repeatability coefficient confirms that absolute differences fall within a 95% probability, while a value above the repeatability coefficient is explained by true changes rather than measurement errors. For tumors an increase or decrease of > 18.1% and for organs a change > 23.5% were assumed to be clinically relevant [34].

## Results

### PBPK model evaluation

**Fig. 1:**
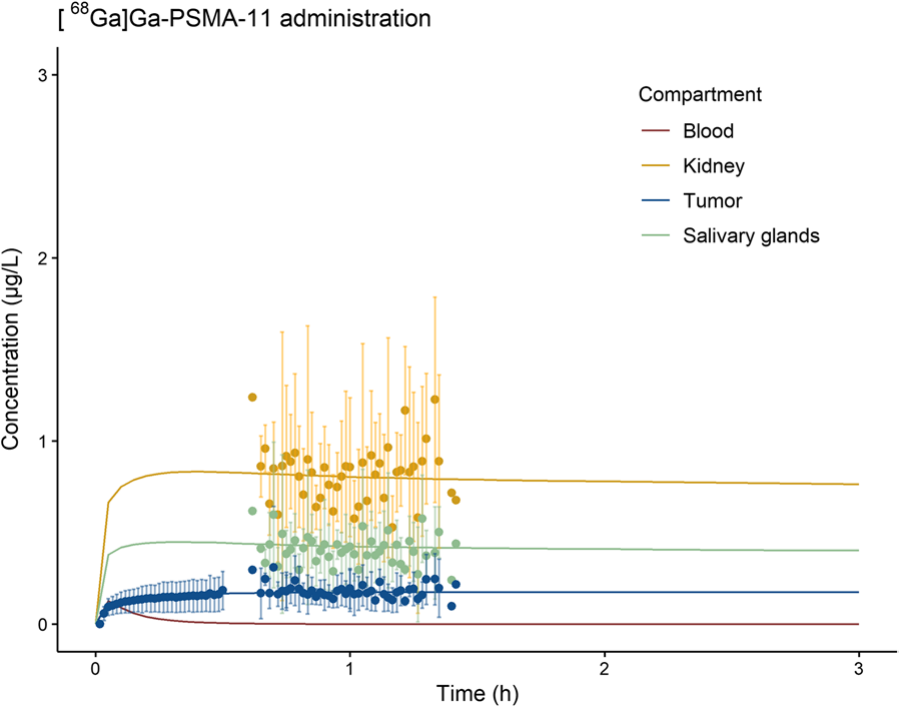
[^68^Ga]Ga-PSMA-11 concentration–time model predictions (solid lines) for blood, salivary glands, kidney and tumor compared to patient observations in similar compartments (dots with standard deviations)

Concentration–time predictions for [^68^Ga]Ga-PSMA-11 based on the final PBPK model are shown in Fig. 1. Model results show predictions for a typical patient, representing the median of this population. Comparing predictions for kidney, salivary glands and tumor with observed patient data, the model adequately predicted data from a typical patient. The results of the sensitivity analysis are provided in Table 2 and showed that the developed model was not highly reliant on particular input parameters.

**Table 2:** Sensitivity analysis results for the salivary glands, kidney and tumor compartments with area under the concentration–time curve (0–24 h) as the output parameter

Compartment	Input parameter	Sensitivity value
Salivary glands	[^68^Ga]Ga-PSMA-11 dose (µg)	0.935
Salivary glands	Salivary glands volume	− 0.901
Salivary glands	PSMA amount salivary glands	0.891
Salivary glands	*K*_D_	− 0.620
Salivary glands	Plasma protein scale factor	0.550
Salivary glands	Fraction unbound	0.550
Kidney	[^68^Ga]Ga-PSMA-11 dose (µg)	0.975
Kidney	Kidney volume	− 0.972
Kidney	PSMA amount kidney	0.693
Kidney	*K*_D_	− 0.519
Tumor	[^68^Ga]Ga-PSMA-11 dose (µg)	1.01
Tumor	Tumor blood flow	0.971

Only sensitivity values < − 0.5 or > 0.5 were reported

*K*_D_, equilibrium dissociation constant; PSMA, prostate-specific membrane antigen

### Effect of folate on [^68^Ga]Ga-PSMA-11 accumulation

**Fig. 2:**
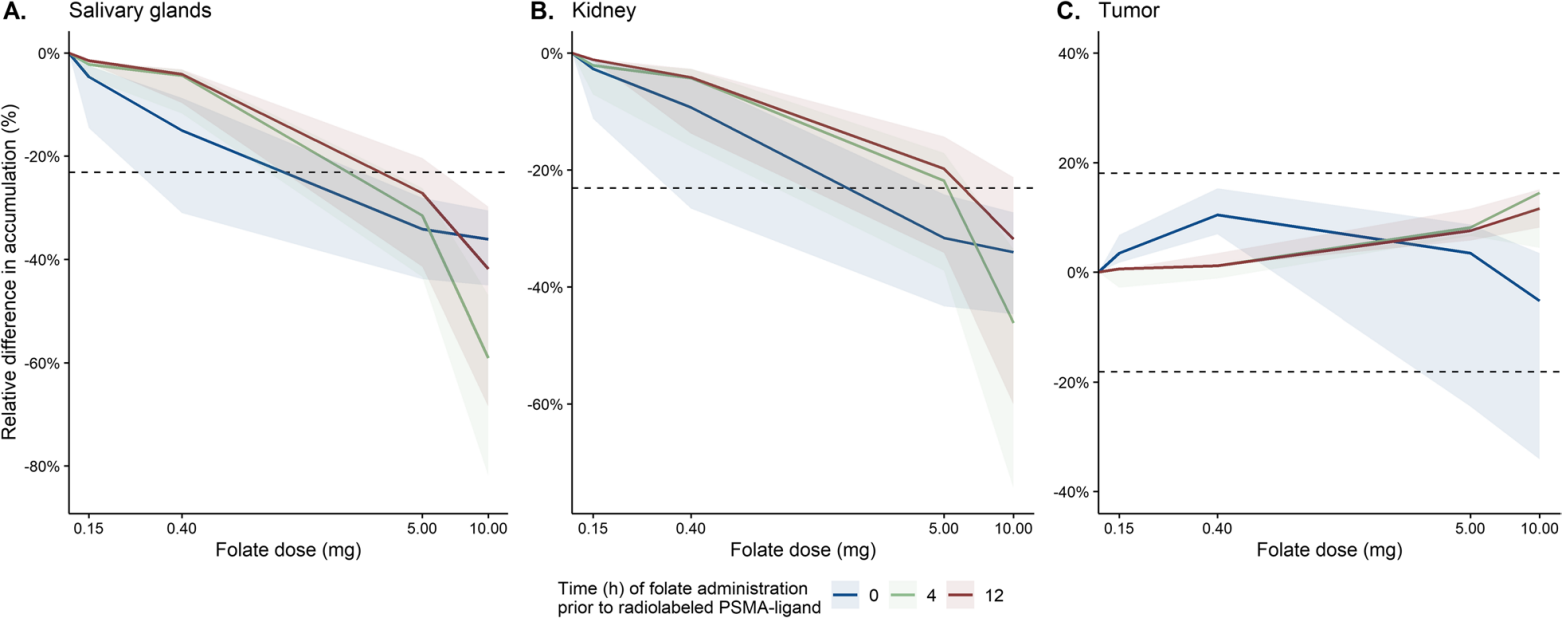
Simulation results showing the effect of different folate doses on [^68^Ga]Ga-PSMA-11 uptake in salivary glands (**A**), kidney (**B**) and tumor (**C**) for different timings of folate administrations. Prediction intervals are caused by ranges in affinity of folate for the PSMA receptor, and dashed lines represent recovery coefficients to clarify clinical relevance of predicted effects

Simulations were performed to compare organ and tumor uptake (µg/L) in situations with and without prior folic acid administration. The results of the simulations are provided in Fig. 2, where prediction intervals were based on different affinities of folate for the PSMA receptor. Simulation results showed a minor relative decrease in uptake in salivary glands and kidney uptake after 150 µg 5-MTHF administration (at *t* = 0; i.e., co-administration) of 5% (range 2–15%) and 3% (range 1–11%), respectively, while uptake in tumors increased with 3% (range 2–7%). Intake of 400 µg folic acid, representing vitamin supplements, resulted in a somewhat larger decrease for salivary glands and kidney (15% and 9% decrease, respectively) and larger increase for tumor (10% increase). With high doses of folic acid (5 mg and 10 mg) at *t* = 0, the folate effect on [^68^Ga]Ga-PSMA-11 accumulation was most profound and clinically relevant, with almost no differences between 5 mg of 10 mg doses. Salivary glands uptake decreased with 34% (range 28–44%) and 36% (range 31–45%) for 5 mg and 10 mg administered doses, respectively, while for kidney the decrease was 32% (24–43%) and 34% (range 27–45%), respectively. Tumor uptake increased with 3% (range − 24 to 9%) and decreased with 5% (range − 34 to 3%) after administration of 5 mg and 10 mg, respectively.

### Timing of folate intake

The timing of folate intake prior to [^68^Ga]Ga-PSMA-11 PET/CT also seemed to play an important role. For folate administration at 4 and 12 h prior to [^68^Ga]Ga-PSMA-11 compared to *t* = 0, the effects of folate intake on [^68^Ga]Ga-PSMA-11 uptake in salivary glands and kidney were smaller with administration of 150 µg 5-MTHF and 400 µg and 5 mg folic acid. However, after 10 mg folic acid dosing, there was a clinical relevant effect on the organ accumulation that, especially 4 h prior to [^68^Ga]Ga-PSMA-11, was even stronger compared to folic acid intake at the same time as [^68^Ga]Ga-PSMA-11 (*t* = 0). For tumors, after high folic acid intake (5 and 10 mg) 4 and 12 h prior to [^68^Ga]Ga-PSMA-11, a slight but clinically irrelevant increase in tumor uptake was predicted.

### Tumor volume differences

**Fig. 3:**
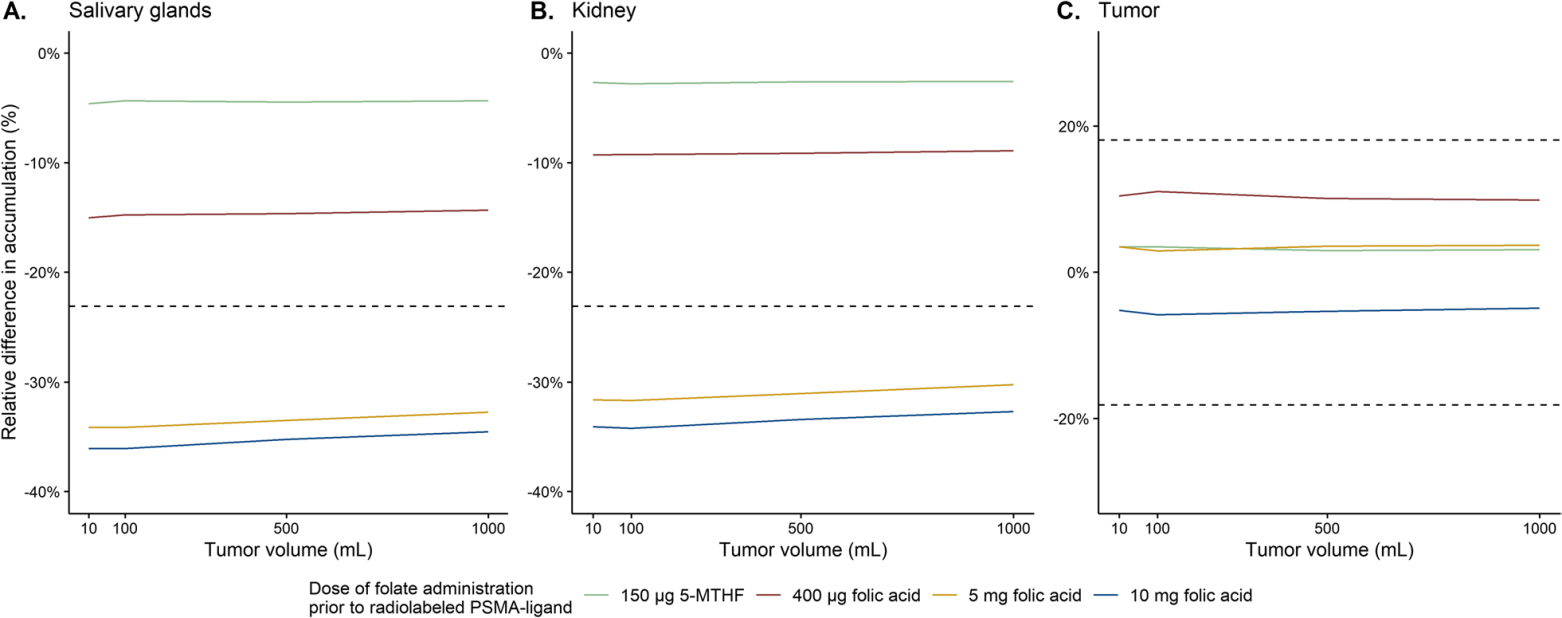
Simulation results showing the effect of different tumor volumes on relative differences in [^68^Ga]Ga-PSMA-11 accumulation caused by different folate doses in salivary glands (**A**), kidney (**B**) and tumor (**C**), where dashed lines represent recovery coefficients to clarify clinical relevance of predicted effects

Changes in accumulation after folic acid and 5-MTHF administration at *t* = 0 were determined for different tumor volumes (10, 100, 500 and 1000 mL), and the results are shown in Fig. 3. Tumor volume only slightly affected the impact of folates on [^68^Ga]Ga-PSMA-11 accumulation in organs and tumors. For example, after 5 mg folic acid administration salivary glands uptake decreased 34% for a patient with 10 mL tumor volume, while for a tumor volume of 1000 mL the decrease was 33%.

## Discussion

PBPK model simulations of folate intake together with [^68^Ga]Ga-PSMA-11 (*t* = 0) showed clinically relevant decreases in salivary glands and kidney uptake only with high folate doses (5 and 10 mg), while effects of folate on tumor uptake were not clinically relevant for all simulated doses. In all cases, folate intake by means of folate containing food (150 µg) or vitamin supplements (400 µg) did not have a relevant impact on [^68^Ga]Ga-PSMA-11 accumulation in salivary glands, kidneys and tumors.

An important issue associated with predicted effects from a PBPK model is the dependency of predictions on the underlying input parameters. During the sensitivity analysis, the input parameters were varied with 10%, while uncertainty in some parameters might exceed 10%. Model predictions for folate effects will be highly dependent on the folate affinity for the PSMA receptor. Therefore, a more extreme range in *K*_D_ was taken into account in model simulations (1–10.8 nM). Unfortunately, the exact affinity of folic acid and 5-MTHF for the PSMA receptor is unknown, while prediction intervals (see Fig. 2) proved the importance of this input value for predictions of the folate effect. The prediction range especially becomes larger for the largest decreases in relative accumulation (i.e., stronger folate effects). This is explained by the fact that in the case of a low impact, the folate concentrations are too low to achieve any effect and predictions are thus barely dependent on the affinity for the PSMA receptor. Conversely, in cases where folate does impact [^68^Ga]Ga-PSMA-11 accumulation (especially at higher folic acid doses) the impact of affinity for the PSMA receptor is more important and predictions are very reliant on affinity input values.

Final PBPK model predictions for [^68^Ga]Ga-PSMA-11 showed accumulation plateaus in the organs of interest at ~ 20 min post-injection. Wen et al*.* reported plateaus reached in liver at ~ 10 min post-injection, while for kidneys and salivary glands uptake increased up to (at least) 60 min post-injection [35]. For kidneys, this could be due to an increase of urine content containing radioactivity over time, while in our predictions [^68^Ga]Ga-PSMA-11 in urine was not part of simulated concentration–time profiles for the whole kidney organ. In this way, we were able to show the effect of folate administration on the PSMA receptor-mediated uptake. For salivary glands, the difference between reported times of the reached plateau and our predictions was less apparent. However, the salivary glands uptake mechanism was assumed similar to other organs (regarding receptor binding and internalization), while it has been suggested that this mechanism might be partly non-PSMA specific [36, 37]. Afshar-Oromieh et al*.* showed that for most patients uptake in salivary glands does not increase any further after 60 min post-injection [38]. Therefore, the plateau reached in salivary glands in our predictions is probably only slightly earlier compared to reported uptake, suggesting that non-PSMA receptor-mediated uptake does not play a major role in salivary glands exposure.

To put the predictions of the folate effect on organ uptake into perspective, these were compared to published clinical results. In our study, co-administration of 10 mg folic acid (at *t* = 0) resulted in a 36% (range 31–45%) decrease of [^68^Ga]Ga-PSMA-11 uptake in salivary glands and 34% (range 27–45%) decrease in kidneys, which is rather comparable to decreases reported by Armstrong et al*.* (46% and 52% decrease in SUV_mean_ for salivary glands and kidneys, respectively) and Harsini et al*.* (26–42% and 28% decrease in SUL_mean_ for all glands and kidneys, respectively) [10, 11]. Rousseau et al*.* also showed a reduced uptake in salivary glands and kidney, without affecting tumor uptake, after MSG administration in a preclinical setting [39]. In addition, Rousseau et al*.* reported a clear dose-dependent effect in mice (MSG dose range 164–657 mg/kg), while our findings only implied a dose-dependent effect in organs up to a folate dose of 5 mg (for administration at *t* = 0). Differences between mice and humans, for example in receptor expressions and renal folate clearance, could explain these different findings regarding dose dependency of the effects.

Predicted effects of high doses folic acid on the normal organs and tumors should be perceived with some considerations. The larger predicted decrease in organ uptake after a 10 mg dose compared to 5 mg at time points 4 h and 12 h prior to [^68^Ga]Ga-PSMA-11 could be due to an underestimation of renal clearance of folate. The fraction of the folate dose excreted renally increases with increased dosing [40], but the PBPK model was not evaluated for 10 mg folate administrations due to lack of patient data. Therefore, the extrapolation to 10 mg in our predictions should be interpreted with some caution. Still, results for 10 mg at *t* = 0 showed that no clear additional effect on organ uptake was predicted compared to 5 mg, which reflected maximum folate effects (probably due to full occupancy of the receptors). However, at the same time point, a decrease in tumor uptake was observed after 10 mg compared to 5 mg folate, which implied that tumor uptake will further reduce with higher folate doses. PCa, and especially metastatic lesions, show a clear overexpression of PSMA receptors compared to healthy human tissues. Higher folate doses will eventually also induce total receptor saturation in tumor lesions, and hence, reduced [^68^Ga]Ga-PSMA-11 uptake in the tumors as a result of competitive binding. This would also clarify differences in our findings for tumor uptake compared to previously published results [10, 11], where tumor uptake of PSMA ligands was significantly reduced after MSG intake. Armstrong et al*.* reported a decrease of 38% in SUV_mean_ after 150 mg/kg MSG, while Harsini et al*.* observed a 29% decrease in SUL_mean_ after 21.7 g MSG [10, 11]. Extrapolating our model simulations to a folic acid intake of 36.5 g (comparable to MSG doses in the prospective studies), tumor uptake indeed decreased even further with a relative difference of 15% (range 2–42%). Therefore, based on our predictions as well as previously published patient studies, caution is warranted with dose selection in case of using folate administration to reduce organ uptake and doses > 10 mg possibly could negatively affect tumor uptake. On the other hand, low doses (< 400 µg) are not expected to affect [^68^Ga]Ga-PSMA-11 biodistribution at all.

The timing of folate intake also impacts the effect on biodistribution, as the decrease in organ uptake seemed more profound in case of 10 mg 4 h prior to [^68^Ga]Ga-PSMA-11 (59% decrease for salivary glands and 46% decrease for kidney) compared to co-administration (*t* = 0). As already discussed, predictions with 10 mg folic acid should be interpret with caution, but still these findings could be explained by a delayed maximum plasma concentration of folates (especially the metabolite 5-MTHF) after oral ingestion of folic acid. However, this increased folate effect seemed diminished with administration 12 h prior to [^68^Ga]Ga-PSMA-11, due to the relatively high clearance of the folate metabolites from the systemic circulation [41].

Radioligand accumulation is even more crucial when considering PSMA-based radionuclide therapy, as accumulation in salivary glands, bone marrow and kidneys is known to induce dose-dependent toxicities and insufficient tumor uptake can lead to a reduced therapy efficacy. Unfortunately, direct translation of our simulations to predict the effects of folate intake on [^177^Lu]Lu-PSMA remains challenging. The main difference between those ligands is the total administered peptide amount, which is an almost 50-fold higher during therapy. Thus, a probable assumption is that also larger folate doses are needed to compete with PSMA ligands for the PSMA receptor during therapy. Another approach to reduce organ uptake by competing for the PSMA receptor could be to add a cold PSMA ligand during the administration of the radiolabeled PSMA ligand. A dose of 5 mg folic acid (0.0103 mmol) would then be comparable to, for example, administration of 9.75 mg PSMA-11 [42], which is somewhat higher compared to the highest cold PSMA-11 mass that was suggested by Kalidindi et al*.* (5.30 mg) [43]. However, one major difference that needs to be considered is that PSMA-11 probably has an increased affinity to the PSMA receptor compared to folates, resulting in a more profound effect, and thus, more prominent reductions in organ and tumor uptake. In case one would design a prospective trial with folate intake prior to [^68^Ga]Ga-PSMA-11, we would recommend a rather low dose of 5 or 10 mg folic acid (at *t* = 0). Minor or negligible effects on tumor uptake are expected, while organ uptake is probably reduced in a clinically relevant manner. Potential dose extrapolations could be performed while examining and evaluating effects on organ and tumor uptake, since a decrease in tumor uptake might be expected.

## Conclusions

Predictions using our final PBPK model showed that co-administration of high doses folate (5 and 10 mg folic acid) with [^68^Ga]Ga-PSMA-11 showed clinically relevant decreased uptake in salivary glands (34% and 36% for 5 and 10 mg, respectively) and kidney (32% and 34% for 5 and 10 mg, respectively) for patients with PCa. No relevant effects of folate administration on tumor uptake were predicted with folate doses ranging from 150 µg to 10 mg, but higher folic acid doses might reduce tumor uptake as well. In addition, model predictions showed that intake by means of folate containing food or vitamin supplements will not affect [^68^Ga]Ga-PSMA-11 biodistribution, independent of the timing of folate intake. Lastly, inter-patient differences in tumor volume did not impact folate effects on [^68^Ga]Ga-PSMA-11 organ uptake. These in silico findings suggest that folate administration prior to [^177^Lu]Lu-PSMA radioligand therapy might be an effective approach to reduce normal organ uptake without affecting tumor accumulation.

## Supplementary Information

**Additional file 1**. Model development and evaluation of the folate PBPK model.

## Data Availability

The datasets used for the current study are available from the corresponding author on reasonable request.
