# Supplementary material for: Predicting the effect of different folate doses on [68Ga]Ga-PSMA-11 organ and tumor uptake using physiologically based pharmacokinetic modeling
Source: EJNMMI Res. 2023 Jun 15;13:60. doi: 10.1186/s13550-023-01008-y (PMC10272068; doi:10.1186/s13550-023-01008-y)
Supplement: Supplementary file 1 — Additional file 1. Model development and evaluation of the folate PBPK model. [file 13550_2023_1008_MOESM1_ESM.pdf]

Additional File 1

Predicting the effect of different folate doses on [^{68}Ga]Ga-PSMA-11 organ and tumor uptake using physiologically based pharmacokinetic modelling

Hinke Siebinga[1,2], Jeroen J.M.A. Hendrikx[1,2], Alwin D.R. Huitema[1,3,4], Berlinda J. de Wit-van der Veen[2]

1 Department of Pharmacy & Pharmacology, The Netherlands Cancer Institute, Amsterdam, The Netherlands

2 Department of Nuclear Medicine, The Netherlands Cancer Institute, Amsterdam, The Netherlands

3 Department of Clinical Pharmacy, University Medical Center Utrecht, Utrecht University, Utrecht, The Netherlands

4 Department of Pharmacology, Princess Máxima Center for Pediatric Oncology, Utrecht, The Netherlands

Corresponding author: Hinke Siebinga, h.siebinga@nki.nl, The Netherlands Cancer Institute, Plesmanlaan 121, 1066 CX Amsterdam, The Netherlands

PBPK model development for folate

Folic acid was used as the compound to represent folate intake. A simplified description of the metabolism was included because of model simplicity, where folic acid was metabolized by one dummy enzyme to one main metabolite 5-methylhydrofolate (5-MTHF). This dummy enzyme is further referred to as dihydrofolate reductase (DHFR), which is the main enzyme responsible for folic acid metabolism. Concentration-time profiles of folic acid and 5-MTHF after oral administration of folic acid (400 µg and 5 mg) were available from literature [1, 2]. Also for 5-MTHF, plasma concentration-time plots after administration of oral 5-MTHF (436 µg and 5 mg) were obtained from literature [1, 2]. Both a low and high dose input were evaluated, to capture two important processes, namely the saturation of the metabolizing enzyme and the increased unchanged folic acid excretion with higher dosages (due to exceeding renal capacity for reabsorption) [3-7]. Data was obtained by using PlotDigitizer (version 3) [8] and these data was used to eventually evaluate and optimize model predictions.

Input parameters for folic acid and 5-MTHF were based on literature values. The molecular weight of folic acid was 441.4 g/mol, while lipophilicity was -2.5 [9]. For 5-MTHF these parameters were 459.5 g/mol and -0.5, respectively. Binding to plasma proteins for folic acid was fixed to 50%, while for 5-MTHF this was 60% [10, 11]. Renal clearance of 5-MTHF was optimized using parameter optimization based on 5-MTHF observations after 5-MTHF dosing. For folic acid renal clearance was scaled to 50% fraction excreted in urine after dosing of 5 mg, assuring that with low doses only a small amount appeared in the urine [12]. The Proton-Coupled Folate Transporter (PCFT), Reduced Folate Carrier (RFC), Multidrug Resistance Proteins 3 (MRP3), Folate Receptor α (FOLR) and Organic Anion Transporter K1 (OAT-K1) were incorporated in the PBPK model to describe active absorption, efflux, transmembrane transport, renal excretion and tubular reabsorption. Transporter locations and transporter-related parameters were initially based on literature and parameters were optimized based on the obtained plasma data [13, 14]. A built-in Monte Carlo algorithm was used for parameter identification to optimize selected input parameters based

on observed patient data. Initial and optimized affinity parameters and the reference concentrations for these transporters are provided in Table S1.

PBPK model evaluation

Model predictions were evaluated for both 5-MTHF and folic acid administration in low and high dose. For 5-MTHF, plasma concentration-time predictions accurately described observed concentrations after both low (436 µg) and high (5 mg) oral dose administration (see Figure S1A). For folic acid, both parent and metabolite concentrations were predicted and evaluated for the low dose (400 µg), while for high dose (5 mg) only metabolite data was available for evaluation. Model predictions after folic acid administration showed adequate description of the observed patient data derived from literature (see Figure S1B), although predictions show a slightly higher and earlier peak in concentration for folic acid.

Table S1 Overview of most important input parameters that were fixed or fitted to describe folic acid and 5-MTHF biodistribution using the PBPK model.

	Fixed or fitted (*) value	Reference
Reference concentration		
- PCFT	1.56 µmol/L *	
- RFC	3.59 µmol/L *	
- MRP3	2.52 µmol/L*	
- FOLR	2.65 nmol/L *	
- OAT-K1	1.00 µmol/L	
- DHFR	0.04 µmol/L *	
K_m (folic acid)		
- PCFT	1.00 µmol/L	[15]
- FOLR1	1.00 nmol/L	[7, 16]
- MRP3	0.0501 mmol/L *	[17]
- OAT-K1	1.00 µmol/L	
- DHFR	0.05 µmol/L *	[18]
V_{max} (folic acid)		
- PCFT	2.49 µmol/L/min *	
- FOLR1	2.90 nmol/mL/min	[7, 16]
- MRP3	36.4 nmol/mL/min *	[17]
- OAT-K1	2.00 µmol/L/min *	
- DHFR	0.02 nmol/mL/min	[18]
K_m (5-MTHF)		
- RFC	1.00 µmol/L	[17, 19]
- FOLR1	10 nmol/L	[7, 16]
- MRP3	0.422 µmol/L *	[17, 19]
V_{max} (5-MTHF)		
- RFC	1.29 µmol/L/min *	[19]
- FOLR1	2.90 nmol/mL/min	[7, 16]
- MRP3	0.0222 nmol/mL/min *	[17]
Renal clearance 5-MTHF	1.22 mL/min/kg *	

Abbreviations: 5-MTHF: 5-methyltetrahydrofolate; FOLR: folate receptor α; K_m *: Michaelis-Menten constant; MRP3: multidrug resistance protein 3; OAT-K1: organic anion transporter K1; PCFT: proton-coupled folate transporter; RFC: reduced folate carrier;* V_{max}*: maximum velocity of the enzymatic reaction.*

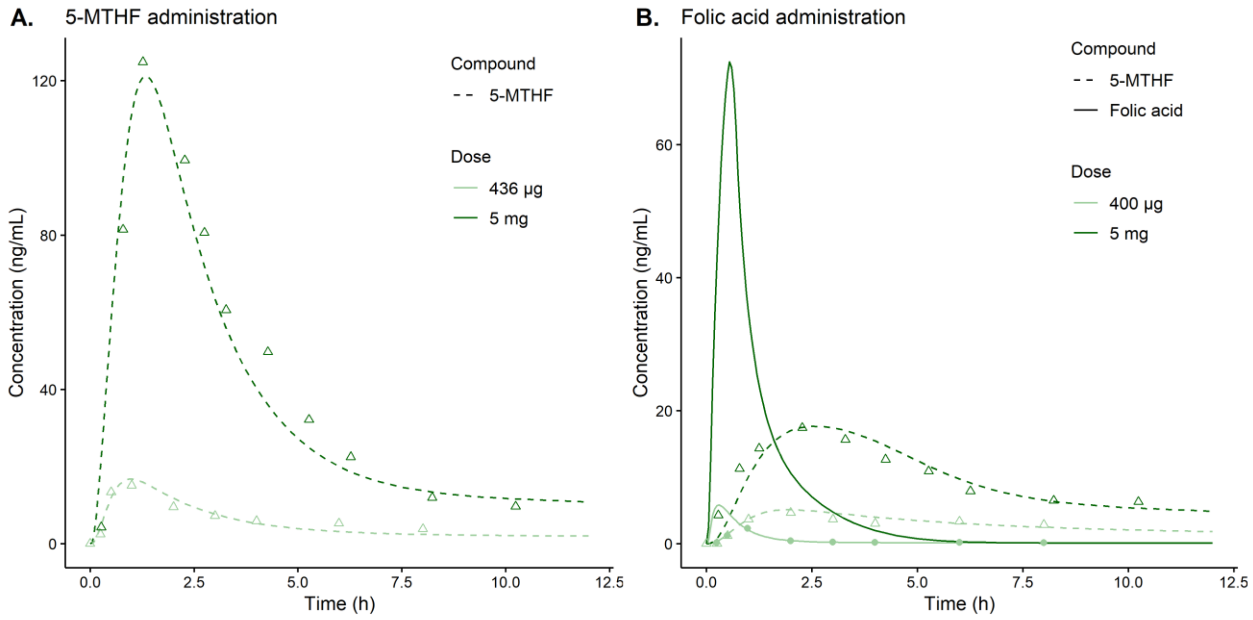

Fig. S1 Concentration-time predictions (lines) vs observations (dots and triangles) for 5-MTHF and folic acid after administration of a low (light green) and high dose (dark green) of both 5-MTHF (A) and folic acid (B).

References

1. Obeid R, Schön C, Pietrzik K, Menzel D, Wilhelm M, Smulders Y, et al. Pharmacokinetics of Sodium and Calcium Salts of (6S)-5-Methyltetrahydrofolic Acid Compared to Folic Acid and Indirect Comparison of the Two Salts. Nutrients. 2020;12. doi:10.3390/nu12123623.

2. Willems FF, Boers GH, Blom HJ, Aengevaeren WR, Verheugt FW. Pharmacokinetic study on the utilisation of 5-methyltetrahydrofolate and folic acid in patients with coronary artery disease. Br J Pharmacol. 2004;141:825-30. doi:10.1038/sj.bjp.0705446.

3. Tam C, O'Connor D, Koren G. Circulating unmetabolized folic Acid: relationship to folate status and effect of supplementation. Obstet Gynecol Int. 2012;2012:485179. doi:10.1155/2012/485179.

4. Bailey SW, Ayling JE. The extremely slow and variable activity of dihydrofolate reductase in human liver and its implications for high folic acid intake. Proc Natl Acad Sci U S A. 2009;106:15424-9. doi:10.1073/pnas.0902072106.

5. Zhao R, Diop-Bove N, Visentin M, Goldman ID. Mechanisms of membrane transport of folates into cells and across epithelia. Annu Rev Nutr. 2011;31:177-201. doi:10.1146/annurev-nutr-072610-145133.

6. Zhao R, Matherly LH, Goldman ID. Membrane transporters and folate homeostasis: intestinal absorption and transport into systemic compartments and tissues. Expert Rev Mol Med. 2009;11:e4. doi:10.1017/s1462399409000969.

7. Samodelov SL, Gai Z, Kullak-Ublick GA, Visentin M. Renal Reabsorption of Folates: Pharmacological and Toxicological Snapshots. Nutrients. 2019;11. doi:10.3390/nu11102353.

8. PlotDigitizer. Available from: https://sourceforge.net/projects/plotdigitizer/. Accessed 15-08-2022.

9. National Center for Biothechnology Information. PubChem Compound Summary for CID 135398658, Folic Acid. 2023. Accessed 12-10-2022.

10. Elsborg L. Binding of folic acid to human plasma proteins. Acta Haematol. 1972;48:207-12. doi:10.1159/000208460.

11. Mader RM, Steger GG, Rizovski B, Djavanmard MP, Scheithauer W, Jakesz R, et al. Stereospecific pharmacokinetics of rac-5-methyltetrahydrofolic acid in patients with advanced colorectal cancer. Br J Clin Pharmacol. 1995;40:209-15.

12. Drugbank. Folic acid. 2005. Available from: https://go.drugbank.com/drugs/DB00158. Accessed 03-10-2022.

13. Patanwala I, King MJ, Barrett DA, Rose J, Jackson R, Hudson M, et al. Folic acid handling by the human gut: implications for food fortification and supplementation. Am J Clin Nutr. 2014;100:593-9. doi:10.3945/ajcn.113.080507.

14. Zamek-Gliszczynski MJ, Sangha V, Shen H, Feng B, Wittwer MB, Varma MVS, et al. Transporters in Drug Development: International Transporter Consortium Update on Emerging Transporters of Clinical Importance. Clin Pharmacol Ther. 2022;112:485-500. doi:10.1002/cpt.2644.

15. Zhao R, Aluri S, Goldman ID. The proton-coupled folate transporter (PCFT-SLC46A1) and the syndrome of systemic and cerebral folate deficiency of infancy: Hereditary folate malabsorption. Mol Aspects Med. 2017;53:57-72. doi:10.1016/j.mam.2016.09.002.

16. Low PS, Antony AC. Folate receptor-targeted drugs for cancer and inflammatory diseases. Adv Drug Deliv Rev. 2004;56:1055-8. doi:10.1016/j.addr.2004.02.003.

17. Zeng H, Chen ZS, Belinsky MG, Rea PA, Kruh GD. Transport of methotrexate (MTX) and folates by multidrug resistance protein (MRP) 3 and MRP1: effect of polyglutamylation on MTX transport. Cancer Res. 2001;61:7225-32.

18. Whitsett J, Rangel Filho A, Sethumadhavan S, Celinska J, Widlansky M, Vasquez-Vivar J. Human endothelial dihydrofolate reductase low activity limits vascular tetrahydrobiopterin recycling. Free Radic Biol Med. 2013;63:143-50. doi:10.1016/j.freeradbiomed.2013.04.035.

19. Ifergan I, Jansen G, Assaraf YG. The reduced folate carrier (RFC) is cytotoxic to cells under conditions of severe folate deprivation. RFC as a double edged sword in folate homeostasis. J Biol Chem. 2008;283:20687-95. doi:10.1074/jbc.M802812200.
